# Symptoms Prior to Diagnosis of Multiple Sclerosis in Individuals Younger Than 18 Years

**DOI:** 10.1001/jamanetworkopen.2024.52652

**Published:** 2024-12-27

**Authors:** Manas K. Akmatov, Jonas Graf, Claudia Kohring, David Ellenberger, Jörg Bätzing, Helen Tremlett, Jakob Holstiege

**Affiliations:** 1 Department of Epidemiology and Health Care Atlas, Central Research Institute of Ambulatory Health Care, Berlin, Germany; 2 Department of Neurology, Medical Faculty and University Hospital Düsseldorf, Heinrich-Heine-University Düsseldorf, Germany; 3 MS Research and Project Development GmbH, Hannover, Germany; 4 Department of Medicine, Division of Neurology, University of British Columbia, Vancouver, British Columbia, Canada; 5 Djavad Mowafaghian Centre for Brain Health, University of British Columbia, Vancouver, British Columbia, Canada

## Abstract

**Importance:**

A growing body of literature suggests the presence of a prodromal period with nonspecific signs and symptoms before onset of multiple sclerosis (MS).

**Objective:**

To systematically assess diseases and symptoms diagnosed in the 5 years before a first MS- or central nervous system (CNS) demyelinating disease–related diagnostic code in pediatric patients compared with controls without MS and controls with another immune-mediated disorder, juvenile idiopathic arthritis (JIA).

**Design, Setting, and Participants:**

This population-based, matched case-control study included children and adolescents (aged <18 years) in Germany with statutory health insurance from January 2010 to December 2020. The study population consisted of 3 groups: case individuals with MS, control individuals without MS, and control individuals with JIA. Data were analyzed from November 2023 to April 2024.

**Exposures:**

Diagnoses coded according to the *International Statistical Classification of Diseases and Related Health Problems, 10th Revision, German Modification (ICD-10-GM)*.

**Main Outcome and Measures:**

The main outcome was incident cases of MS, defined as the first confirmed diagnosis of MS (*ICD-10-GM* code G35) in 1 quarter between 2013 and 2020 and at least 1 additional diagnosis in the following quarters. In total, 163 *ICD-10-GM* codes before a first MS diagnosis were assessed using univariable and multivariable logistic regression analyses.

**Results:**

The study population consisted of 1091 children and adolescents with MS, 10 910 without MS, and 1068 with JIA. Of the children and adolescents with MS, 788 (72.2%) were female. Mean (SD) age at disease diagnosis was 15.7 (1.7) years. Nine *ICD-10-GM* codes were present more frequently among children and adolescents with MS in the 5 years before their first MS diagnosis than among controls without MS: obesity (adjusted odds ratio [AOR], 1.70; 95% CI, 1.42-2.02), disorders of eye refraction and accommodation (AOR, 1.26; 95% CI, 1.09-1.47), visual disturbances (AOR, 1.31; 95% CI, 1.10-1.55), gastritis and duodenitis (AOR, 1.35; 95% CI, 1.08-1.70), patella disorders (AOR, 1.47; 95% CI, 1.13-1.90), heartbeat abnormalities (AOR, 1.94; 95% CI, 1.27-2.96), flatulence (AOR, 1.43; 95% CI, 1.01-2.01), skin sensation disturbances (AOR, 12.93; 95% CI, 8.98-18.62), as well as dizziness and giddiness (AOR, 1.52; 95% CI, 1.22-1.89). Four of these *ICD-10-GM* codes were significantly more prevalent in children and adolescents with MS than in controls with JIA: obesity (AOR, 3.19; 95% CI, 2.03-5.02), refraction and accommodation disorders (AOR, 3.08; 95% CI, 2.33-4.08), visual disturbances (AOR, 1.62; 95% CI, 1.13-2.33), and skin sensation disturbances (AOR, 27.70; 95% CI, 6.52-117.64).

**Conclusions and Relevance:**

In this population-based, matched case-control study, children and adolescents with MS had diverse metabolic, ocular, musculoskeletal, gastrointestinal, and cardiovascular symptoms, signs, or diagnoses within 5 years before their first MS diagnosis. Better characterization of early symptoms and/or risk factors, comorbid disorders, and possible prodromal features of MS may have considerable implications for early recognition and subsequent progression of the disease.

## Key Points

**Question:**

Do certain diseases, signs, or symptoms occur in the 5 years before a first diagnosis of multiple sclerosis (MS) in pediatric patients?

**Findings:**

In this case-control study of 1091 children and adolescents (aged <18 years) with MS and matched controls, 9 disorders were present significantly more frequently in the 5 years before a first MS diagnosis than in controls: obesity, disorders of eye refraction and accommodation, visual disturbances, gastritis and duodenitis, patella disorders, heartbeat abnormalities, flatulence, skin sensation disturbances, as well as dizziness and giddiness.

**Meaning:**

The findings suggest that children and adolescents with MS have diverse metabolic, ocular, musculoskeletal, gastrointestinal, and cardiovascular symptoms, signs, and diseases within 5 years before their first MS diagnosis.

## Teaser

This case-control study investigates whether certain diseases or symptoms occur more often in children and adolescents with multiple sclerosis (MS) in the 5 years before diagnosis than in control individuals.

## Introduction

Multiple sclerosis (MS) is a chronic immune-mediated disease of the central nervous system (CNS) characterized by demyelination, with manifestation most commonly occurring in adulthood. Typically, MS manifests between the ages of 20 and 30 years. However, up to 10% of all patients with MS are younger than 18 years at first manifestation, and less than 1% are younger than 10 years.^1^ A growing body of research suggests that signs or symptoms nonspecific for MS manifest years before disease onset, suggestive of a prodromal phase.^2,3^ For example, a Canadian study showed that patients with MS had higher utilization of both inpatient and outpatient medical care in the 5 years before a first demyelinating event compared with matched general population controls.^4,5^ The main reasons for health care utilization were diseases of the nervous system, musculoskeletal system, sensory organs, diseases of the blood, and mental health problems.^5^ A higher frequency of medication use, such as antivertigo medications, antiepileptics, glucocorticoids, urinary antispasmodics, and muscle relaxants, in the 5 years before a first demyelinating event in patients with MS compared with controls was observed in another Canadian study.^6^ In addition, visual disturbances, musculoskeletal disorders, and disturbances of skin sensation were more frequently diagnosed before a first demyelinating event related to MS.^6^

Less is known about the prodromal features in pediatric MS. A population-based study from Canada showed that the proportion of children hospitalized in the year before initial MS diagnosis was higher among children and adolescents with MS than among controls.^7^ Using nationwide German medical claims data, we designed a matched case-control study to systematically assess diseases and symptoms diagnosed in the 5 years before a first MS diagnosis in pediatric patients.

## Methods

### Data Source

In this case-control study, we analyzed nationwide German outpatient claims data for all individuals with statutory health insurance (SHI) from January 2010 to December 2020. The SHI includes nearly 88% of the total German population. These data are reported by SHI-authorized physicians on a quarterly basis for billing purposes. The dataset comprised outpatient diagnoses coded using the *International Statistical Classification of Diseases and Related Health Problems, 10th Revision, German Modification (ICD-10-GM)*^8^ along with diagnostic certainty (eg, confirmed or excluded) and demographic characteristics (sex, age, and region of residence) of insured individuals who visited an SHI-authorized physician at least once during the study period. The use of claims data for scientific research is regulated by the Social Security Code in Germany. Ethical approval and informed consent were not required, as this study used routinely collected anonymized data. The research was conducted in accordance with the Declaration of Helsinki.^9^ The study followed the Strengthening the Reporting of Observational Studies in Epidemiology (STROBE) reporting guideline.

### Study Design and Population

We designed a matched case-control study to identify diseases and signs or symptoms diagnosed in the 5 years before a first MS diagnosis. Due to the nationwide coverage of the data and the relatively long period, this dataset was appropriate to study early risk factors and comorbidities as well as prodromal features for various chronic conditions.^10,11,12^ Case ascertainment was conducted annually in a cohort of pediatric patients younger than 18 years (n = 10 715 794 in 2020) with outpatient visits in the year of ascertainment and without an MS diagnosis in the preceding 3 years. Incident cases were defined as a first confirmed diagnosis of MS (*ICD-10-GM* code G35) in 1 quarter between 2013 and 2020 and at least 1 additional diagnosis in 1 of the following quarters (eFigure 1 in Supplement 1). One of the MS diagnoses had to have been coded by a neurologist. Length of individual follow-up before a first diagnosis of MS varied between 3 and 5 years and depended on the time of first MS diagnosis, calendar years available in the dataset (2010-2020), and the first year with an outpatient visit (eFigure 1 in Supplement 1). The latter was 2010 for most cases. eFigure 2 in Supplement 1 describes the ascertainment of children and adolescents with MS. The index quarter was the earliest recorded MS-related or CNS demyelinating disease–related code (CNS inflammatory or demyelinating diseases [G04, G05, G09, G35, G36, or G37], optic nerve or visual pathway disorders [H46, H47, or H48], or abnormal findings on diagnostic CNS imaging [R90]).^13^ Children and adolescents with a first CNS demyelinating disease–related code only (ie, without a confirmed MS diagnosis) were not defined as having MS. Controls without an MS- or CNS-related demyelinating disease diagnosis were randomly matched to each case (10:1) by sex, age at first MS diagnosis (in years), region of residence (represented by the 17 regional Associations of Statutory Health Insurance Physicians), and observation period. In addition, we selected a second control group of children and adolescents newly diagnosed with juvenile idiopathic arthritis (JIA), another chronic immune-mediated disorder of different pathophysiology.^14^ Including an additional control group of patients with another autoimmune disorder was done to provide further information as to whether any observed associations were specific to MS or also common in other autoimmune diseases. Ascertainment of incident JIA cases was conducted using a similar approach as for incident MS cases; children or adolescents had to have received the first confirmed diagnosis of JIA (*ICD-10-GM* code M08.8) in 1 quarter between 2013 and 2020 and another confirmed diagnosis in a subsequent quarter. For the comparison of children and adolescents with MS and JIA, matching variables were identical to matching with controls without MS, but the target case-to-control ratio was 1:1.

### Exposure

We performed exploratory analysis by examining the frequency of confirmed diagnoses in the 5 years before a first MS diagnosis. Diagnoses made in the penultimate quarter (preindex) were not considered in the analysis as they may reflect the diagnostic workup of MS. Diagnoses from all 22 *ICD-10-GM* chapters were included in the analysis using 3-digit codes. Among the 1222 possible 3-digit codes, to adhere to the data protection and access agreements, codes with fewer than 30 children or adolescents with MS and/or controls were not included, leaving 163 *ICD-10-GM* codes for analyses (eTable 1 in Supplement 1).

### Statistical Analysis

Initially, we examined the association of 163 *ICD-10-GM* codes with a later MS diagnosis by calculating univariable odds ratios (ORs) and corresponding 95% CIs for the 5-year period. *P* values were corrected for multiple testing by using the Šidák correction.^15^ Furthermore, we performed multivariable analysis by including all *ICD-10-GM* codes that remained statistically significant after the Šidák correction in a logistic regression model also adjusted for sex and age. In addition to the pooled analysis for the whole 5-year period, we calculated ORs for each individual year to determine when potential differences in diseases or symptoms between children and adolescents with MS and controls occurred. Furthermore, we applied the same multivariable logistic model for the comparison of children and adolescents with MS and JIA. Finally, we conducted a complementary analysis by removing children and adolescents with neurological or cerebrovascular diagnoses since these disorders may be considered early indicators of MS^13^ (eTable 2 in Supplement 1). The ORs for each disease, sign, and symptom in this analysis were adjusted for sex and age, as balancing between cases and controls was removed due to exclusion of children and adolescents with neurological or cerebrovascular diagnoses. This analysis was done using separate logistic regression models. Analyses were performed from November 2023 to April 2024 using SAS for Windows, version 9.4 (SAS Institute Inc), and R, version 4.0.5 (R Project for Statistical Computing). Two-sided *P* <.05 was considered significant.

## Results

### Description of the Study Populations

**Figure 1.**
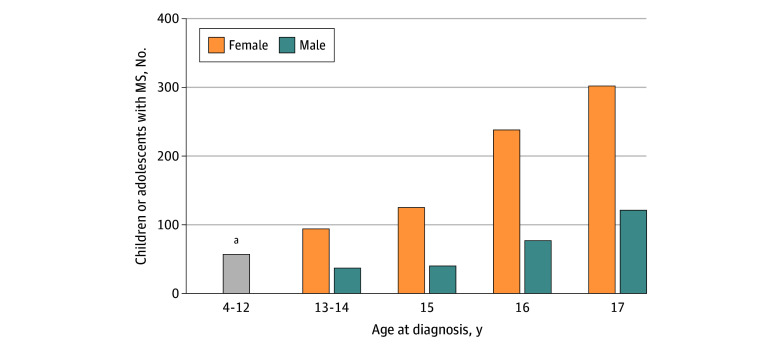
Distribution of Children and Adolescents With Newly Diagnosed Multiple Sclerosis (MS) by Sex and Age ^a^The sex distribution could not be depicted due to data protection regulations.

The study population consisted of 1091 children or adolescents with MS, 10 910 without MS, and 1068 with JIA (Table). The majority of children or adolescents with MS (933 [85.5%]) had a first diagnosis of MS (*ICD-10-GM* code G35) in the index quarter, and the remainder (158 [14.5%]) had a CNS-related demyelinating disease code that was coded before the first MS diagnosis. Another 24 individuals with incident cases (2.2%) experienced their first demyelinating event before January 1, 2013, and were excluded from the study population due to insufficient observation time before the index quarter (eFigure 2 in Supplement 1). Of the 1091 children and adolescents included, 788 (72.2%) were female and 303 (27.8%) were male (Table). Figure 1 shows the distribution of children and adolescents with MS by sex and age. The number of children and adolescents with MS was lowest in the age group of 4 to 12 years and increased in each consecutive age group, with the mean (SD) age at the index quarter being 15.7 (1.7) years. The control groups without MS and with JIA included 10 910 and 1068 children and adolescents, respectively (Table). For the comparison of MS vs JIA, 23 children or adolescents with MS (2.1%) were excluded, as a match with JIA could not be found.

**Table.** Description of the Study Populations

Characteristic	Patients with MS (n = 1091)	Controls without MS (n = 10 910)	Controls with JIA (n = 1068)^a^
Sex, No. (%)			
Female	788 (72.2)	7880 (72.2)	776 (72.7)
Male	303 (27.8)	3030 (27.8)	292 (27.3)
Age at first diagnosis, mean (SD), y	15.7 (1.7)	15.7 (1.7)	15.7 (1.7)
Follow-up time, mean (SD), y^b^	4.1 (1.1)	4.1 (1.1)	4.1 (1.1)

Abbreviations: JIA, juvenile idiopathic arthritis; MS, multiple sclerosis.

^a^ For 23 children or adolescents with MS, a match with JIA was not found. These children and adolescents were excluded from the comparative analysis of MS and JIA.

^b^ Time from first recorded outpatient visit during the 5-year observation period to the index date.

### *ICD-10-GM* Codes Before a First MS Diagnosis or Demyelinating Event

**Figure 2.**
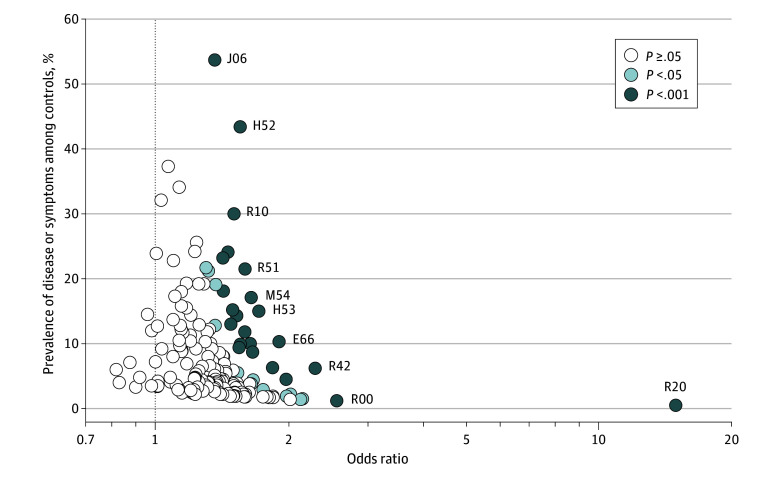
Univariable Odds Ratios for Diseases and Symptoms Among Individuals With Multiple Sclerosis (MS) Compared With Controls by Prevalence Among Controls According to Significance Level Each circle represents an examined disease or symptom defined by the first 3 characters of an *International Statistical Classification of Diseases and Related Health Problems, 10th Revision, German Modification (ICD-10-GM)* code: E66, obesity; H52, disorders of refraction and accommodation; H53, visual disturbances; J06, acute upper respiratory infections of multiple and unspecified sites; M54, dorsalgia; R00, abnormalities of heartbeat; R10, abdominal and pelvic pain; R20, disturbances of skin sensation; R42, dizziness and giddiness; and R51, headache. In total, 163 *ICD-10-GM* codes that could be analyzed without violation of data protection regulations are depicted. The control group was matched by sex, age, region of residence, and observation period. Odds ratios and 95% CIs are provided in eTable 3 in Supplement 1. *P* values were adjusted by the Šidák correction.^15^

**Figure 3.**
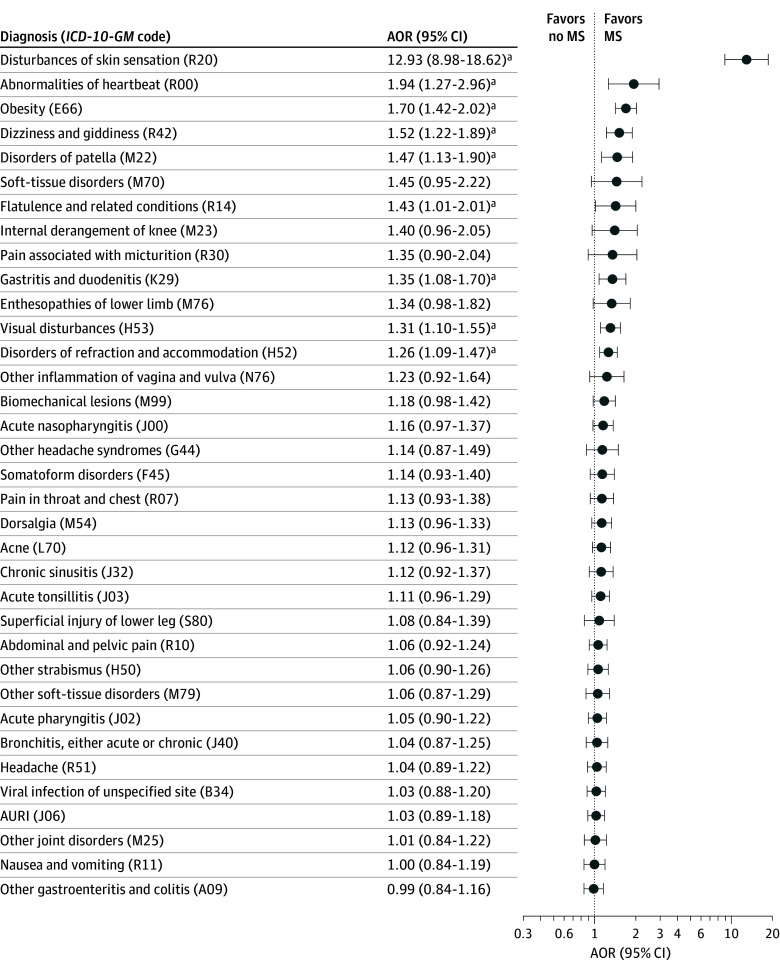
Adjusted Odds Ratios (AORs) of Multiple Sclerosis (MS) Diagnosis in Patients With MS Compared With Controls by Diseases and Symptoms ORs were adjusted for all other *International Statistical Classification of Diseases and Related Health Problems, 10th Revision, German Modification (ICD-10-GM)* codes in eTable 3 in Supplement 1 as well as sex and age. Control groups were matched by sex, age, region of residence, and observation period. AURI indicates acute upper respiratory infections of multiple and unspecified sites. ^a^Significant result.

Of the 163 *ICD-10-GM* codes assessed, 96 (58.9%) were present more frequently among children and adolescents with MS in the 5 years before the MS diagnosis than among controls at the significance level of 5%. After adjustment for multiple testing, the difference in frequency of 35 of those codes (36.4%) remained statistically significant (Figure 2 and eTable 3 in Supplement 1). The codes comprised various diseases, including infectious diseases and diseases of the eye, respiratory system, digestive system, and musculoskeletal system as well as various other symptoms, signs, and abnormal clinical and laboratory findings. The prevalence of these diagnoses in the control group ranged from 0.5% (disturbances of skin sensation [*ICD-10-GM* code R20]) to 53.7% (acute upper respiratory infections of multiple and unspecified sites [code J06]). In a multivariable model including all 35 codes plus sex and age, 9 of the disease codes (25.7%) remained associated with a later MS diagnosis (Figure 3 and eTable 3 in Supplement 1). These codes were for obesity (E66) (adjusted OR [AOR], 1.70; 95% CI, 1.42-2.02); 2 eye disorders, including disorders of refraction and accommodation (H52) (AOR, 1.26; 95% CI, 1.09-1.47) and visual disturbances (H53) (AOR, 1.31; 95% CI, 1.10-1.55); 1 gastrointestinal disorder (gastritis and duodenitis [K29]) (AOR, 1.35; 95% CI, 1.08-1.70); 1 musculoskeletal disorder (disorders of patella [M22]) (AOR, 1.47; 95% CI, 1.13-1.90); and 4 codes from the *ICD-10-GM* classification “symptoms, signs and abnormal clinical and laboratory findings, not elsewhere classified,” including abnormalities of heartbeat (R00) (AOR, 1.94; 95% CI, 1.27-2.96), flatulence and related conditions (R14) (AOR, 1.43; 95% CI, 1.01-2.01), disturbances of skin sensation (R20) (AOR, 12.93; 95% CI, 8.98-18.62), and dizziness and giddiness (R42) (AOR, 1.52; 95% CI, 1.22-1.89) (Figure 3 and eTable 3 in Supplement 1). Of these, 4 disorders (44.4%) were still significantly associated with a later MS diagnosis when comparing children and adolescents with MS with those with JIA: obesity (AOR, 3.19; 95% CI, 2.03-5.02), disorders of refraction and accommodation (AOR, 3.08; 95% CI, 2.33-4.08), visual disturbances (AOR, 1.62; 95% CI, 1.13-2.33), and disturbances of skin sensation (AOR, 27.70; 95% CI, 6.52-117.64) (eTable 3 in Supplement 1). Children and adolescents with 1 of 3 musculoskeletal diagnoses—other joint disorders (M25), other soft-tissue disorders (M79), and biomechanical lesions (M99)—were less likely to be diagnosed with MS compared with children and adolescents with JIA.

In the complementary analysis, we excluded 702 children and adolescents (64.3%) from the group with MS and 4131 (37.9%) from the control group without MS due to neurological or cerebrovascular diagnoses that may be considered early indicators of MS in the 5 years before the index quarter. Overall, 4 of the 35 diseases or symptoms (11.4%) that were significantly more frequently reported before a first MS diagnosis in the main analysis were excluded.^13^ Of the remaining 31 diseases, signs, or symptoms, 8 (25.8%) remained significantly more frequent in a multivariable analysis (eTable 3 in Supplement 1). Of note, 2 disorders (6.5%), obesity and disorders of refraction and accommodation, were associated with later MS in all 3 comparisons groups (eTable 3 in Supplement 1).

**Figure 4.**
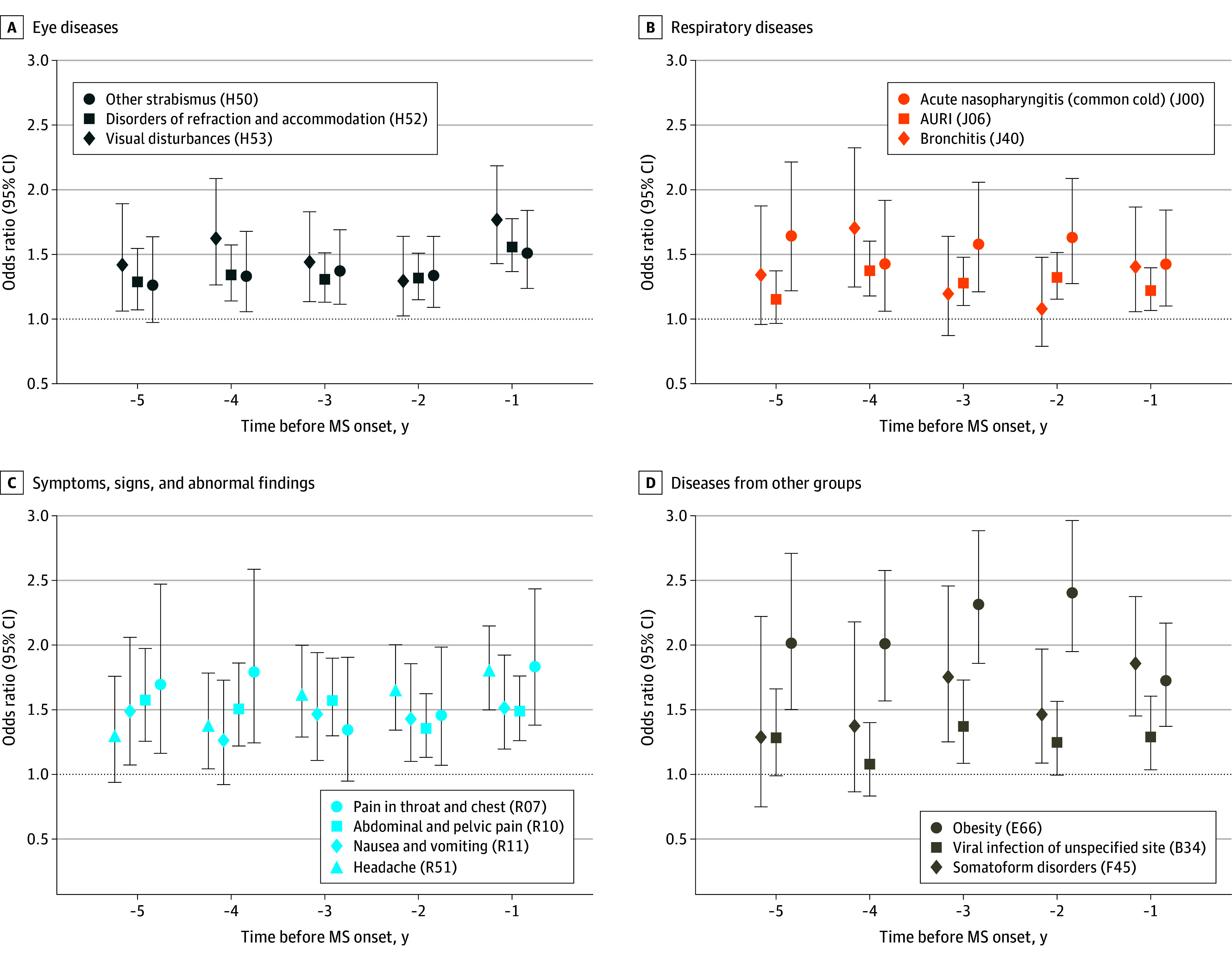
Univariable Odds Ratios of Multiple Sclerosis (MS) for Selected Diseases and Symptoms in the 5 Years Before a First MS Diagnosis for Each Year in the Preobservational Period by Disease Group *International Statistical Classification of Diseases and Related Health Problems, 10th Revision, German Modification (ICD-10-GM) *codes are shown in parentheses. Whiskers represent 95% CIs. AURI indicates acute upper respiratory infections of multiple and unspecified sites.

Figure 4 shows the ORs of MS for selected diseases, signs, and symptoms for each of the 5 years before a first MS diagnosis. For most of the diseases, signs, and symptoms, there were higher odds of MS within the 5 years before the index quarter. There was no trend in ORs over the 5 years for any of the diseases and symptoms.

## Discussion

Taking advantage of a comprehensive nationwide dataset containing outpatient diagnoses of all children and adolescents with SHI in Germany, we applied an exploratory approach to systematically examine diseases and/or symptoms diagnosed years before a first MS- or CNS-related demyelinating diagnosis. This is of particular importance since better characterization of early symptoms and/or risk factors, comorbid disorders, and possible prodromal features of MS may have considerable implications for early recognition of MS and subsequent progression of the disease.^16^ To our knowledge, such a systematic investigation has never been done in pediatric populations. Due to the nationwide coverage of the data included, we were able to include a large sample of children and adolescents with MS (n = 1091), which in turn allowed us to detect associations with small and medium effect sizes. This is of particular importance as pediatric MS is a rare disorder. Furthermore, we used 2 control groups, children and adolescents without MS and those with another autoimmune disorder, JIA. The inclusion of the latter group may have allowed exclusion of associations that could have been attributed to inflammatory processes and thus may not have been due to MS-specific pathophysiological mechanisms.

In the main analysis, after sex and age adjustments, we found 9 disorders, signs, and symptoms significantly more frequently diagnosed 5 years before a first MS diagnosis than in children and adolescents without MS. These included obesity (*ICD-10-GM* code E66), 1 gastrointestinal disorder (K29), 1 musculoskeletal disorder (M22), 2 disorders of the eye (H52 and H53), and 4 unspecific diagnoses (R00, R14, R20, and R42). Two disorders, obesity and disorders of refraction and accommodation, remained significantly positively associated with MS in the complementary analysis (when children and adolescents with other neurological and cerebrovascular disorders were excluded) and compared with children and adolescents with JIA. The disorder with the highest AOR was disturbances of skin sensation, followed by abnormalities of heartbeat and obesity. The remaining disorders had small effect sizes, with AORs ranging between 1.26 (95% CI, 1.09-1.47) (disorders of refraction and accommodation) and 1.52 (95% CI, 1.22-1.89) (dizziness and giddiness).^17^

Obesity and, to a lesser extent, overweight in childhood^18,19,20^ and early adulthood^20^ are known risk factors for the development of MS. Sex-specific differences in the risk of MS have been observed; extreme obesity before MS was significantly associated with the risk of MS and/or clinically isolated syndrome in girls but not boys.^18^ There was no association of overweight and moderate obesity with risk of MS in that study, possibly due to the low sample size in that study (n = 75). More recently, Milles et al^19^ observed a positive association between a higher body mass index (BMI) and MS in both females and males. A causal role of obesity in the development of MS was observed in a large-scale mendelian randomization study; the risk of MS increased by 41% for each 1-unit increase in the SD of genetically determined BMI.^21^ Our finding regarding the presumed exposure to obesity before first MS diagnosis is in line with these studies. Of note, children and adolescents with a diagnosis of obesity already present 5 years before first MS diagnosis had a 2-fold higher risk of later being diagnosed with MS than children and adolescents without obesity. There are several pathophysiological explanations for this association.^20^ First, obesity represents a condition characterized by low-grade inflammation, which may contribute to the development of neuroinflammation.^22^ Furthermore, several adipokines with both proinflammatory and anti-inflammatory functions have been linked to autoimmune disorders, including MS.^23^ In addition, obesity is associated with a lower serum 25-hydroxyvitamin D level, which in turn has been shown to be a risk factor for MS.^24^

In the main analysis, we found 4 unspecific diagnoses from chapter XVIII [R] of the *ICD-10-GM* classification “symptoms, signs and abnormal clinical and laboratory findings, not elsewhere classified”; similar findings have been observed by others.^5,13^ Diagnoses made from this chapter are usually less defined, can be of unknown etiology, and require additional diagnostic procedures to make a more specific diagnosis. The unspecific nature of these symptoms, signs, and diagnoses may be indicative of possible prodromal features of MS. In fact, the diagnosis disturbances of skin sensation (R20) had the largest effect size in this study (AOR, 12.93; 95% CI, 8.98-18.62). This AOR was double when using children and adolescents with JIA as a control group (AOR, 27.70; 95% CI, 6.52-117.64). These findings are in agreement with findings from other studies.^5,25^ For example, Wijnands et al^5^ observed that patients with relapsing-onset MS were more likely to visit dermatologists in the 5 years preceding MS onset than were patients without MS. Other unspecific diagnoses for which findings were significant in the main analysis were abnormalities of heartbeat (R00), dizziness and giddiness (R42), and flatulence and related conditions (R14). Given their low background prevalence (ie, prevalence among controls) and a relatively high AOR for MS, these diagnoses may help to facilitate an early diagnosis of MS. Thus, it would be worthwhile to raise awareness among general practitioners who see pediatric patients years before an initial diagnosis of MS.

Both cardiovascular and gastrointestinal symptoms have been reported in patients with MS.^5,26,27^ Cardiovascular dysfunction, including orthostatic intolerance, dysfunctions of heart rate and rhythm, and left ventricular dysfunction, may be part of the clinical picture of MS.^26^ In addition, evidence of early gastrointestinal symptoms before initial MS diagnosis exists.^28^ In a retrospective cohort study over 14 years, Almeida et al^28^ observed that every third adult patient with MS had gastrointestinal symptoms before their first demyelination event. The most frequent gastrointestinal symptoms or disorders reported were constipation (50%), diarrhea (30%), and irritable bowel syndrome (17%). In that study, the first manifestation of gastrointestinal symptoms was observed at an average 3.7 years before a first demyelination event. Yusuf et al^29^ found higher rates of physician visits due to gastritis and duodenitis and diseases of the esophagus in the 5 years before a first demyelinating event or MS onset. We found several diagnoses related to the gastrointestinal system, including gastroenteritis and colitis of infectious and unspecified origin (A09), gastritis and duodenitis (K29), and 2 unspecific diagnoses (abdominal and pelvic pain [R10] and nausea and vomiting [R11]), of which gastritis and duodenitis remained positively associated with MS after controlling for other variables.

Visual impairment is one of the frequent complaints in patients of all ages with MS.^30^ Every third child with MS has optic neuritis as the initial presenting symptom.^31^ In the main univariable analysis, we found 3 diagnoses positively associated with a later MS diagnosis, of which 2—disorders of refraction and accommodation (H52) and visual disturbances (H53)—remained significantly associated with later MS diagnosis in the multivariable analysis. The diagnosis code H53 comprises a range of subjective disorders, including amblyopia and subjective visual disturbances, and may indicate the presence of visual impairment. In line with our findings, another German study found higher rates of visual disturbances 5 years before an MS diagnosis (in a cohort of patients without a clinically isolated syndrome).^13^ To our knowledge, the association of a more specific diagnosis (H52) with a later MS diagnosis has never been observed. However, a study showed that the accommodative ability is diminished in patients with MS compared with healthy individuals.^32^

Musculoskeletal symptoms belong to the clinical picture of MS, including muscle pain and bone and joint disorders. In addition, a study reported a high prevalence of musculoskeletal comorbidities in patients with MS.^27^ Every seventh patient with a new MS diagnosis had a musculoskeletal disorder in 1 study.^33^ In the univariable analyses, we found 8 diagnoses from the *ICD-10-GM* chapter on musculoskeletal diseases that were significantly associated with MS. Only 1 of them, disorders of patella (M22), was associated with a later MS diagnosis after adjusting the model for all other disorders. Similar findings were observed in other studies; for example, physician and hospital visits due to musculoskeletal disorders were more frequent among patients with MS in the 5 years before MS diagnosis or a first demyelinating event compared with controls.^5^ Of note, children and adolescents with 1 of 3 musculoskeletal diagnoses—other joint disorders, other soft-tissue disorders, and biomechanical lesions—were less likely to be diagnosed with MS compared with children and adolescents with JIA. These diagnoses may be considered early symptoms and/or prodromal features of JIA.

### Limitations

The following limitations of the study should be considered when interpreting the results. First, we used administrative claims data that were primarily collected for billing purposes. Thus, both disease and exposure misclassification cannot be ruled out. In particular, there may have been an increased probability of the detection of some diseases and symptoms in cases compared with controls due to higher health care utilization among cases before their first MS diagnosis.^4,5^ To minimize the possibility of including children and adolescents with a false-positive MS diagnosis, we applied a conservative case definition (ie, at least 2 confirmed diagnoses in 2 different quarters with at least 1 diagnosis coded by a neurologist). The same approach was applied to define exposures (albeit without confirmation by a neurologist). Second, due to the large study size and the number of the examined variables, some of the observed associations may be false positive. To reduce this possibility, we selected a conservative approach by adjusting for multiple tests. Third, the dataset does not include inpatient data and data for persons insured privately.

## Conclusions

In this case-control study, children and adolescents with MS had diverse metabolic, ocular, musculoskeletal, gastrointestinal, and cardiovascular symptoms, signs, and diagnoses within 5 years before MS diagnosis. These diagnoses (eg, sensory and ocular disorders) may be considered early symptoms and overlap with known risk factors (eg, obesity). In addition, unspecific diagnoses of unknown nature may be indicative of prodromal features (eg, disturbances of skin sensation, abnormalities of heartbeat, and flatulence). To our knowledge, this is the first study that systematically examined diagnoses made years before an initial MS diagnosis in a pediatric setting. The study used an exploratory approach to identify possible associations, which need to be validated in future studies. The observed associations may be used to generate hypotheses for future studies.

## Notes

Supplement 1. **eFigure 1.** Data of Pediatric Patients With Newly Diagnosed Multiple Sclerosis (MS) Between 2013 and 2020 Included in the Assessment of Medical Diagnoses Before First Manifestation of MS, With Varying Lengths of Observation Time **eFigure 2.** Flowchart Describing Ascertainment of Children and Adolescents With Multiple Sclerosis **eTable 1.** Description of the 163 *ICD-10-GM* Codes Examined in the Study, Grouped by Chapter **eTable 2.** Neurological and Cerebrovascular Diagnoses Excluded in the Complementary Analysis **eTable 3.** Diseases and Symptoms Present More Frequently Among Children and Adolescents With Multiple Sclerosis Before Its Diagnosis as Compared With Controls Without MS and Controls With JIA

Supplement 2.
Data Sharing Statement

## References

[1] Yan K, Balijepalli C, Desai K, Gullapalli L, Druyts E. Epidemiology of pediatric multiple sclerosis: a systematic literature review and meta-analysis. Mult Scler Relat Disord. 2020;44:102260. doi:10.1016/j.msard.2020.102260 32540746

[2] Makhani N, Tremlett H. The multiple sclerosis prodrome. Nat Rev Neurol. 2021;17(8):515-521. doi:10.1038/s41582-021-00519-3 34155379 PMC8324569

[3] Yusuf FLA, Ng BC, Wijnands JMA, Kingwell E, Marrie RA, Tremlett H. A systematic review of morbidities suggestive of the multiple sclerosis prodrome. Expert Rev Neurother. 2020;20(8):799-819. doi:10.1080/14737175.2020.1746645 32202173

[4] Wijnands JMA, Kingwell E, Zhu F, . Health-care use before a first demyelinating event suggestive of a multiple sclerosis prodrome: a matched cohort study. Lancet Neurol. 2017;16(6):445-451. doi:10.1016/S1474-4422(17)30076-5 28434855

[5] Wijnands JM, Zhu F, Kingwell E, . Five years before multiple sclerosis onset: phenotyping the prodrome. Mult Scler. 2019;25(8):1092-1101. doi:10.1177/1352458518783662 29979093

[6] Zhao Y, Wijnands JMA, Högg T, . Interrogation of the multiple sclerosis prodrome using high-dimensional health data. Neuroepidemiology. 2020;54(2):140-147. doi:10.1159/000505331 31940638

[7] Marrie RA, O’Mahony J, Maxwell CJ, ; Canadian Pediatric Demyelinating Disease Network. High rates of health care utilization in pediatric multiple sclerosis: a Canadian population-based study. PLoS One. 2019;14(6):e0218215. doi:10.1371/journal.pone.0218215 31185042 PMC6559708

[8] *International Statistical Classification of Diseases and Related Health Problems, 10th revision, German Modification (ICD-10-GM)*. Federal Institute for Drugs and Medical Devices. 2019. Accessed October 1, 2021. https://www.dimdi.de/dynamic/en/classifications/icd/icd-10-gm/index.html

[9] World Medical Association. World Medical Association Declaration of Helsinki: ethical principles for medical research involving human subjects. JAMA. 2013;310(20):2191-2194. doi:10.1001/jama.2013.281053 24141714

[10] Akmatov MK, Ermakova T, Bätzing J. Psychiatric and nonpsychiatric comorbidities among children with ADHD: an exploratory analysis of nationwide claims data in Germany. J Atten Disord. 2021;25(6):874-884. doi:10.1177/1087054719865779 31364481

[11] Akmatov MK, Ermakova T, Holstiege J, Steffen A, von Stillfried D, Bätzing J. Comorbidity profile of patients with concurrent diagnoses of asthma and COPD in Germany. Sci Rep. 2020;10(1):17945. doi:10.1038/s41598-020-74966-1 33087813 PMC7578650

[12] Schrag A, Bohlken J, Dammertz L, . Widening the spectrum of risk factors, comorbidities, and prodromal features of Parkinson disease. JAMA Neurol. 2023;80(2):161-171. doi:10.1001/jamaneurol.2022.3902 36342675 PMC9641600

[13] Gasperi C, Hapfelmeier A, Daltrozzo T, Schneider A, Donnachie E, Hemmer B. Systematic assessment of medical diagnoses preceding the first diagnosis of multiple sclerosis. Neurology. 2021;96(24):e2977-e2988. doi:10.1212/WNL.0000000000012074 33903190

[14] Zaripova LN, Midgley A, Christmas SE, Beresford MW, Baildam EM, Oldershaw RA. Juvenile idiopathic arthritis: from aetiopathogenesis to therapeutic approaches. Pediatr Rheumatol Online J. 2021;19(1):135. doi:10.1186/s12969-021-00629-8 34425842 PMC8383464

[15] Šidák Z. Rectangular confidence regions for the means of multivariate normal distributions. J Am Stat Assoc. 1967;62:626-633. doi:10.2307/2283989

[16] Marrie RA, Allegretta M, Barcellos LF, . From the prodromal stage of multiple sclerosis to disease prevention. Nat Rev Neurol. 2022;18(9):559-572. doi:10.1038/s41582-022-00686-x 35840705

[17] Chen H, Cohen P, Chen S. How big is a big odds ratio? interpreting the magnitudes of odds ratios in epidemiological studies. Commun Stat Simul Comput. 2010;39:860-864. doi:10.1080/03610911003650383

[18] Langer-Gould A, Brara SM, Beaber BE, Koebnick C. Childhood obesity and risk of pediatric multiple sclerosis and clinically isolated syndrome. Neurology. 2013;80(6):548-552. doi:10.1212/WNL.0b013e31828154f3 23365063 PMC3589288

[19] Milles P, De Filippo G, Maurey H, Tully T, Deiva K; KidBiosep. Obesity in pediatric-onset multiple sclerosis: a French cohort study. Neurol Neuroimmunol Neuroinflamm. 2021;8(5):8. doi:10.1212/NXI.0000000000001044 34285094 PMC8293287

[20] Schreiner TG, Genes TM. Obesity and multiple sclerosis—a multifaceted association. J Clin Med. 2021;10(12):10. doi:10.3390/jcm10122689 34207197 PMC8234028

[21] Mokry LE, Ross S, Timpson NJ, Sawcer S, Davey Smith G, Richards JB. Obesity and multiple sclerosis: a Mendelian randomization study. PLoS Med. 2016;13(6):e1002053. doi:10.1371/journal.pmed.1002053 27351487 PMC4924848

[22] Novo AM, Batista S. Multiple sclerosis: implications of obesity in neuroinflammation. Adv Neurobiol. 2017;19:191-210. doi:10.1007/978-3-319-63260-5_8 28933066

[23] Correale J, Marrodan M. Multiple sclerosis and obesity: the role of adipokines. Front Immunol. 2022;13:1038393. doi:10.3389/fimmu.2022.1038393 36457996 PMC9705772

[24] Mokry LE, Ross S, Ahmad OS, . Vitamin D and risk of multiple sclerosis: a mendelian randomization study. PLoS Med. 2015;12(8):e1001866. doi:10.1371/journal.pmed.1001866 26305103 PMC4549308

[25] Wijnands JMA, Zhu F, Kingwell E, . Prodrome in relapsing-remitting and primary progressive multiple sclerosis. Eur J Neurol. 2019;26(7):1032-1036. doi:10.1111/ene.13925 30714270

[26] Kaplan TB, Berkowitz AL, Samuels MA. Cardiovascular dysfunction in multiple sclerosis. Neurologist. 2015;20(6):108-114. doi:10.1097/NRL.0000000000000064 26671744

[27] Marrie RA, Reider N, Stuve O, . The incidence and prevalence of comorbid gastrointestinal, musculoskeletal, ocular, pulmonary, and renal disorders in multiple sclerosis: a systematic review. Mult Scler. 2015;21(3):332-341. doi:10.1177/1352458514564488 25538150 PMC4429162

[28] Almeida MN, Silvernale C, Kuo B, Staller K. Bowel symptoms predate the diagnosis among many patients with multiple sclerosis: a 14-year cohort study. Neurogastroenterol Motil. 2019;31(6):e13592. doi:10.1111/nmo.13592 30957307

[29] Yusuf FLA, Zhu F, Evans C, . Gastrointestinal conditions in the multiple sclerosis prodrome. Ann Clin Transl Neurol. 2024;11(1):185-193. doi:10.1002/acn3.51945 38115680 PMC10791028

[30] Dhanapalaratnam R, Markoulli M, Krishnan AV. Disorders of vision in multiple sclerosis. Clin Exp Optom. 2022;105(1):3-12. doi:10.1080/08164622.2021.1947745 34348598

[31] Yeh EA, Chitnis T, Krupp L, ; US Network of Pediatric Multiple Sclerosis Centers of Excellence. Pediatric multiple sclerosis. Nat Rev Neurol. 2009;5(11):621-631. doi:10.1038/nrneurol.2009.158 19826402

[32] Küçük B, Hamamcı M, Aslan Bayhan S, Bayhan HA, Inan LE. Amplitude of accommodation in patients with multiple sclerosis. Curr Eye Res. 2019;44(11):1271-1277. doi:10.1080/02713683.2019.1629596 31172825

[33] Dallmeijer AJ, Beckerman H, de Groot V, van de Port IG, Lankhorst GJ, Dekker J. Long-term effect of comorbidity on the course of physical functioning in patients after stroke and with multiple sclerosis. J Rehabil Med. 2009;41(5):322-326. doi:10.2340/16501977-0335 19363563

