# Supplementary material for: Symptoms Prior to Diagnosis of Multiple Sclerosis in Individuals Younger Than 18 Years
Source: JAMA Netw Open. 2024 Dec 27;7(12):e2452652. doi: 10.1001/jamanetworkopen.2024.52652 (PMC11681376; doi:10.1001/jamanetworkopen.2024.52652)
Supplement: Supplement 1. — eFigure 1. Data of Pediatric Patients With Newly Diagnosed Multiple Sclerosis (MS) Between 2013 and 2020 Included in the Assessment of Medical Diagnoses Before First Manifestation of MS, With Varying Lengths of Observation Time eFigure 2. Flowchart Describing Ascertainment of Children and Adolescents With Multiple Sclerosis eTable 1. Description of the 163 ICD-10-GM Codes Examined in the Study, Grouped by Chapter eTable 2. Neurological and Cerebrovascular Diagnoses Excluded in the Complementary Analysis eTable 3. Diseases and Symptoms Present More Frequently Among Children and Adolescents With Multiple Sclerosis Before Its Diagnosis as Compared With Controls Without MS and Controls With JIA [file jamanetwopen-e2452652-s001.pdf]

Supplementary Online Content

Akmatov MK, Graf J, Kohring C, et al. Symptoms prior to diagnosis of multiple sclerosis in individuals younger than 18 years. *JAMA Netw Open*. 2024;7(12):e2452652. doi:10.1001/jamanetworkopen.2024.52652

eFigure 1. Data of Pediatric Patients With Newly Diagnosed Multiple Sclerosis (MS) Between 2013 and 2020 Included in the Assessment of Medical Diagnoses Before First Manifestation of MS, With Varying Lengths of Observation Time
eFigure 2. Flow Chart Describing Ascertainment of Children and Adolescents With Multiple Sclerosis
eTable 1. Description of the 163 *ICD-10-GM* Codes Examined in the Study, Grouped by Chapter
eTable 2. Neurological and Cerebrovascular Diagnoses Excluded in the Complementary Analysis
eTable 3. Diseases and Symptoms Present More Frequently Among Children and Adolescents With Multiple Sclerosis Before Its Diagnosis as Compared With Controls

This supplementary material has been provided by the authors to give readers additional information about their work.

© 2024 Akmatov MK et al. *JAMA Network Open*.

eFigure 1. Examples of data presentations of pediatric patients with newly diagnosed multiple sclerosis (MS) between 2013 and 2020 included into the assessment of medical diagnoses before first manifestation of MS with varying lengths of observation time depending on the calendar year of first outpatient visit and the time of first MS/CNS-related demyelinating disease diagnoses

* Possible demyelinating events were inflammatory diseases of the CNS (ICD-10 codes G04, G05, G09),

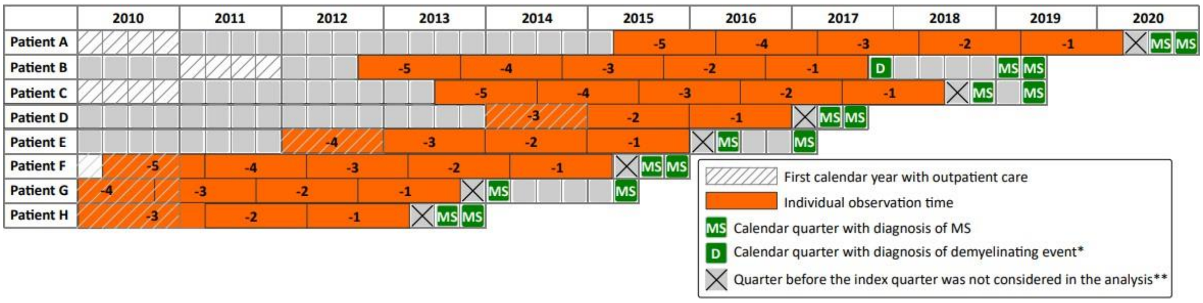

demyelinating diseases of the CNS (G36, G37), disorders of the optic nerve and the visual pathways (H46, H47, H48), and abnormal findings on diagnostic imaging of CNS (R90) [12].

** Diagnoses made in the last quarter before the index quarter were not considered in the analysis as they may not be interpreted as prodromal features of MS but rather be attributed to MS.

© 2024 Akmatov MK et al. *JAMA Network Open*.

eFigure 2. Flow-chart describing the ascertainment of children/adolescents with multiple sclerosis (MS)

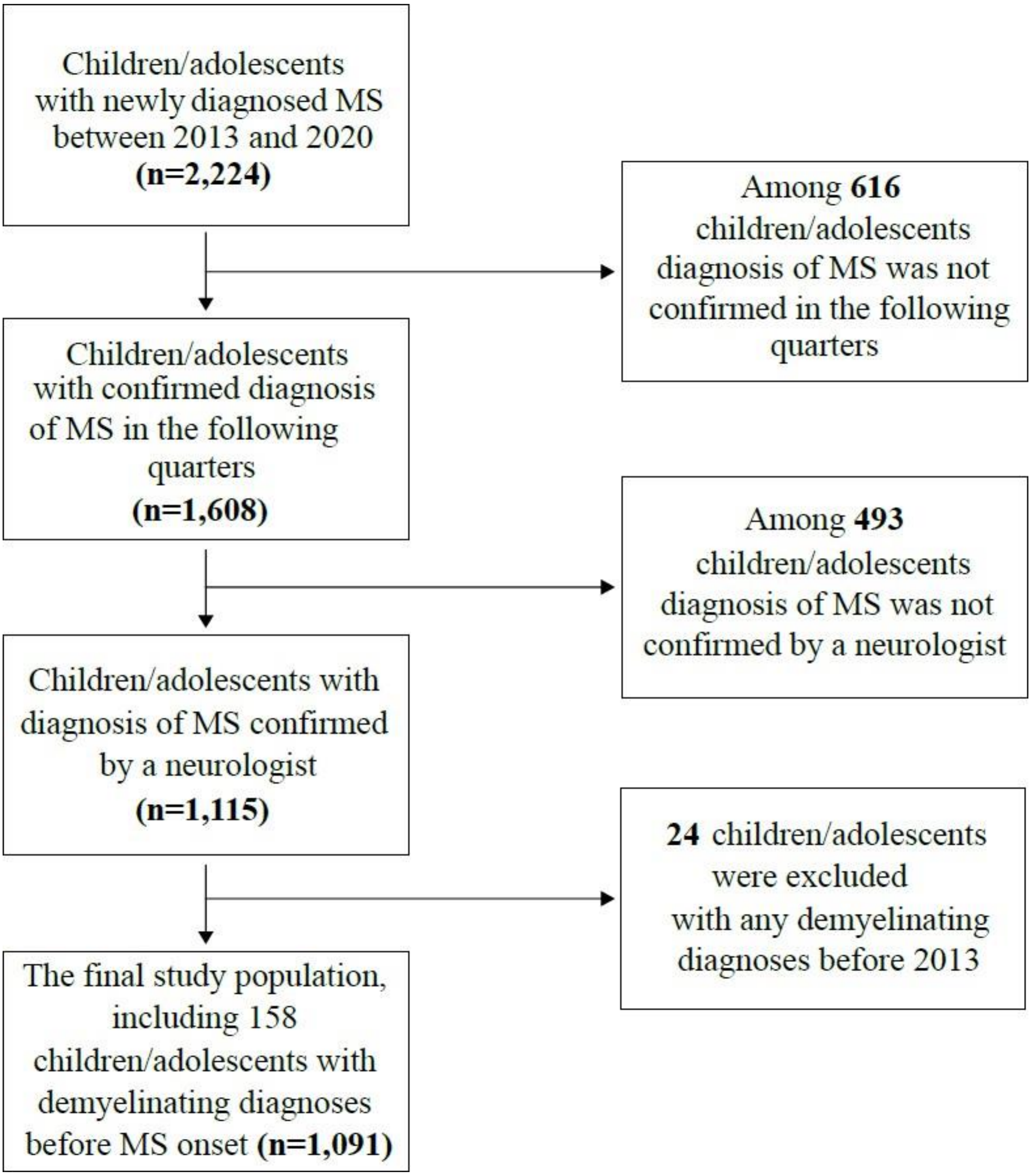

© 2024 Akmatov MK et al. *JAMA Network Open*.

eTable 1. Description of the one hundred sixty-three ICD-10-GM codes examined in the study grouped by ICD chapter

ICD-10-GM chapter	Chapter name	ICD-10 code
I.	Infectious and parasitic diseases	A08, A09, A38, A49, B00, B07, B08, B34, B35, B36, B37, B80, B85, B99
II.	Neoplasms	D22
III.	Diseases of the blood and blood-forming organs	D50
IV.	Endocrine, nutritional and metabolic diseases	E03, E55, E66
V.	Mental and behavioral disorders	F32, F41, F43, F45, F80, F81, F90, F91, F93, F98
VI.	Diseases of the nervous system	G43, G44, G47
VII.	Diseases of the eye and adnexa	H00, H04, H10, H35, H50, H52, H53
VIII.	Diseases of the ear and mastoid process	H60, H61, H65, H66, H68, H69, H91, H92, H93
IX.	Diseases of the circulatory system	I88, I95, I99
X.	Diseases of the respiratory system	J00, J01, J02, J03, J04, J06, J11, J18, J20, J22, J30, J31, J32, J34, J35, J40, J44, J45, J98
XI.	Diseases of the digestive system	K07, K12, K29, K52, K59
XII.	Diseases of the skin and subcutaneous tissue	L01, L02, L03, L08, L20, L23, L29, L30, L50, L60, L65, L70, L72, L85, L90
XIII.	Diseases of the musculoskeletal system and connective tissue	M21, M22, M23, M25, M40, M41, M43, M53, M54, M62, M65, M70, M76, M77, M79, M99
XIV.	Diseases of the genitourinary system	N30, N39, N64, N76, N89, N91, N92, N94
XVII.	Congenital malformations, deformations and chromosomal abnormalities	Q66
XVIII.	Symptoms, signs and abnormal clinical and laboratory findings, not elsewhere classified	R00, R04, R05, R06, R07, R10, R11, R14, R20, R21, R23, R29, R30, R42, R50, R51, R52, R53, R55, R59, R63
XIX.	Injury, poisoning and certain other consequences of external causes	S00, S20, S30, S50, S52, S60, S61, S63, S80, S83, S90, S93, T14, T78, T79
XXI.	Factors influencing health status and contact with health services	Z00, Z01, Z23, Z24, Z25, Z26, Z27, Z30, Z46, Z51, Z63, Z71
One hundred sixty-three diagnoses with the sufficient sample size (at least 30 children/adolescents) were selected for the analysis. Diagnoses with the number of children/adolescents less than 30 (ie, low prevalent diseases) could not be included in the analysis due to the data protection issues.		

© 2024 Akmatov MK et al. *JAMA Network Open*.

eTable2. Neurological and cerebrovascular diagnoses excluded in the complementary analysis

Diagnosis name	ICD-10-GM code(s)
diseases of the nervous system	G00-G99, except G35
cerebrovascular diseases	I60-I69
visual disturbances	H53
disorders of vestibular function	H81
neuromuscular dysfunction of bladder, not elsewhere classified	N31
disturbances of skin sensation	R20
abnormalities of gait and mobility	R26
unspecified urinary incontinence	R32
other unspecified symptoms and signs involving the urinary system	R39
dizziness and giddiness	R42
speech disturbances, not elsewhere classified	R47
Children/adolescents with these diagnoses were excluded in a complementary analysis as they can be considered early indicators of MS.	

eTable 3. Diseases/symptoms present more frequently among children/adolescents with multiple sclerosis (MS) before its diagnosis as compared to controls without MS and controls with JIA – results of the logistic regression analyses

				MS vs. controls without MS (main analysis)					MS vs. controls with JIA (main analysis)		MS vs. controls without MS (complementary analysis)	
**ICD-10 code**	**Diagnosis name**	**Patients with MS**	**Controls without MS**	**Univariable analysis**			**Multivariable analysis****		**Multivariable analysis****		**Multivariable analysis****	
		**n**	**n**	**UOR**	**95% CI**	**p value***	**AOR**	**95% CI**	**AOR**	**95% CI**	**AOR**	**95% CI**
A09	Other gastroenteritis and colitis of infectious and unspecified origin	267	2,088	1.37	1.18-1.58	0.004	0.99	0.84-1.16	1.18	0.83-1.67	1.01	0.86-1.18
B34	Viral infection of unspecified site	285	2,308	1.32	1.14-1.52	0.02	1.03	0.88-1.20	1.05	0.76-1.46	1.03	0.89-1.20
E66	Obesity	195	1,120	1.90	1.61-2.25	6.64 x 10^{-12}	**1.70**	**1.42-2.02**	**3.19**	**2.03-5.02**	**1.68**	**1.41-1.99**
F45	Somatoform disorders	149	950	1.66	1.38-2.00	1.40 x 10^{-05}	1.14	0.93-1.40	0.89	0.59-1.35	1.21	0.99-1.47
G44	Other headache syndromes	78	483	1.66	1.30-2.13	0.009	1.14	0.87-1.49	0.91	0.52-1.59	-	-
H50	Other strabismus	261	1,973	1.42	1.23-1.65	0.0004	1.06	0.90-1.26	0.87	0.62-1.23	1.12	0.95-1.33
H52	Disorders of refraction and accommodation	593	4,732	1.55	1.37-1.76	7.38 x 10^{-10}	**1.26**	**1.09-1.47**	**3.08**	**2.33-4.08**	**1.34**	**1.16-1.54**
H53	Visual disturbances	253	1,635	1.71	1.47-1.99	3.48 x 10^{-10}	**1.31**	**1.10-1.55**	**1.62**	**1.13-2.33**	-	-
J00	Acute nasopharyngitis [common cold]	221	1,556	1.53	1.31-1.79	2.08 x 10^{-05}	1.16	0.97-1.37	1.46	0.99-2.13	1.18	0.99-1.39
J02	Acute pharyngitis	327	2,526	1.42	1.24-1.63	8.02 x 10^{-05}	1.05	0.90-1.22	1.21	0.88-1.67	1.05	0.91-1.22
J03	Acute tonsillitis	345	2,626	1.46	1.27-1.67	6.72 x 10^{-06}	1.11	0.96-1.29	**1.66**	**1.21-2.28**	1.13	0.98-1.31
J06	AURI of multiple and unspecified sites	668	5,857	1.36	1.20-1.55	0.0003	1.03	0.89-1.18	**3.59**	**2.76-4.68**	1.04	0.91-1.19
J32	Chronic sinusitis	151	1,027	1.55	1.29-1.86	0.0005	1.12	0.92-1.37	1.09	0.69-1.70	1.12	0.92-1.36
J40	Bronchitis, not specified as acute or chronic	182	1,396	1.37	1.15-1.62	0.048	1.04	0.87-1.25	**1.54**	**1.03-2.30**	1.07	0.90-1.28
K29	Gastritis and duodenitis	120	687	1.84	1.50-2.26	8.98 x 10^{-07}	**1.35**	**1.08-1.70**	1.12	0.68-1.84	**1.36**	**1.09-1.70**
L70	Acne	289	2,365	1.30	1.13-1.50	0.04	1.12	0.96-1.31	**2.16**	**1.57-2.96**	**1.16**	**1.00-1.34**
M22	Disorders of patella	93	492	1.97	1.57-2.49	1.30 x 10^{-06}	**1.47**	**1.13-1.90**	1.12	0.68-1.84	**1.44**	**1.12-1.86**
M23	Internal derangement of knee	40	206	1.98	1.40-2.79	0.02	1.41	0.96-2.05	0.94	0.47-1.88	1.41	0.97-2.04
M25	Other joint disorders, not elsewhere classified	197	1,413	1.48	1.26-1.75	0.0004	1.01	0.84-1.22	**0.40**	**0.28-0.56**	1.03	0.86-1.23
M54	Dorsalgia	277	1,869	1.65	1.42-1.90	2.76 x 10^{-09}	1.13	0.96-1.33	1.10	0.79-1.52	**1.20**	**1.02-1.41**
M70	Soft tissue disorders related to use, overuse and pressure	31	148	2.13	1.44-3.15	0.03	1.45	0.95-2.22	0.77	0.35-1.69	1.43	0.94-2.17
M76	Enthesopathies of lower limb, excluding foot	55	321	1.75	1.31-2.35	0.03	1.34	0.98-1.83	0.72	0.42-1.23	1.32	0.97-1.79
M79	Other soft tissue disorders, not elsewhere classified	159	1,078	1.56	1.30-1.86	0.0002	1.06	0.87-1.29	**0.41**	**0.29-0.58**	1.13	0.94-1.37

© 2024 Akmatov MK et al. *JAMA Network Open*.

M99	Biomechanical lesions, not elsewhere classified	192	1,290	1.59	1.35-1.88	6.74 x 10^{-06}	1.18	0.98-1.42	**0.65**	**0.45-0.93**	**1.20**	**1.00-1.45**
N76	Other inflammation of vagina and vulva	67	417	1.65	1.26-2.15	0.04	1.23	0.92-1.64	**2.04**	**1.05-3.97**	1.23	0.93-1.63
R00	Abnormalities of heart beat	32	127	2.57	1.73-3.80	0.0004	**1.94**	**1.27-2.96**	1.13	0.46-2.79	**1.95**	**1.29-2.95**
R07	Pain in throat and chest	168	1,091	1.64	1.37-1.95	6.21 x 10^{-06}	1.13	0.93-1.38	1.12	0.73-1.72	1.16	0.96-1.41
R10	Abdominal and pelvic pain	428	3,274	1.51	1.32-1.71	6.52 x 10^{-08}	1.06	0.92-1.24	**1.66**	**1.21-2.28**	1.05	0.90-1.21
R11	Nausea and vomiting	230	1,654	1.49	1.28-1.74	5.62 x 10^{-05}	1.00	0.84-1.19	1.09	0.74-1.62	1.06	0.89-1.26
R14	Flatulence and related conditions	47	238	2.02	1.47-2.78	0.003	**1.43**	**1.01-2.01**	1.86	0.81-4.27	1.38	0.99-1.93
R20	Disturbances of skin sensation	78	56	14.92	10.52-21.16	0.0	**12.93**	**8.98-18.62**	**27.70**	**6.52-117.64**	-	-
R30	Pain associated with micturition	34	161	2.15	1.48-3.13	0.01	1.35	0.90-2.04	1.73	0.60-5.02	1.43	0.96-2.12
R42	Dizziness and giddiness	143	673	2.29	1.89-2.78	0.0	**1.52**	**1.22-1.89**	1.45	0.90-2.34	-	-
R51	Headache	332	2,349	1.59	1.39-1.83	3.86 x 10^{-09}	1.04	0.89-1.22	1.19	0.86-1.65	1.12	0.96-1.30
S80	Superficial injury of lower leg	90	604	1.53	1.22-1.93	0.04	1.08	0.84-1.39	0.94	0.56-1.58	1.15	0.90-1.46

* The p values were adjusted by the Šidák correction [14].
** Adjusted for sex, age and all other ICD codes in the table, significant findings in a multivariable analysis are in bold. A OR, adjusted odds ratio; AURI, acute upper respiratory infections; CI, confidence intervals; UOR, unadjusted odds ratio

© 2024 Akmatov MK et al. *JAMA Network Open*.
