# Supplementary material for: Symptoms Prior to Diagnosis of Multiple Sclerosis in Individuals Younger Than 18 Years
Source: JAMA Netw Open. 2024 Dec 27;7(12):e2452652. doi: 10.1001/jamanetworkopen.2024.52652 (PMC11681376; doi:10.1001/jamanetworkopen.2024.52652)
Supplement: Supplement 2. — Data Sharing Statement [file jamanetwopen-e2452652-s002.pdf]

Data Sharing Statement

Akmatov. Symptoms Prior to Diagnosis of Multiple Sclerosis in Individuals Younger Than 18 Years. *JAMA Netw Open*. Published December 27, 2024.
doi:10.1001/jamanetworkopen.2024.52652

Data
Data available: No

Additional Information
Explanation for why data not available: The datasets analysed during the current study are not publicly available due to data protection regulations by the German Social Security Code (Sozialgesetzbuch V).
